# PKM2 promotes tumor angiogenesis by regulating HIF-1α through NF-κB activation

**DOI:** 10.1186/s12943-015-0490-2

**Published:** 2016-01-06

**Authors:** Ninel Azoitei, Alexander Becher, Konrad Steinestel, Arefeh Rouhi, Kristina Diepold, Felicitas Genze, Thomas Simmet, Thomas Seufferlein

**Affiliations:** Center for Internal Medicine I, University of Ulm, Albert-Einstein-Allee 23, 89081 Ulm, Germany; Gerhard-Domagk-Institute of Pathology, University of Münster, 48149 Münster, Germany; Center for Internal Medicine III, University of Ulm, 89081 Ulm, Germany; Institute of Pharmacology of Natural Products & Clinical Pharmacology, University of Ulm, 89081 Ulm, Germany

**Keywords:** Pancreatic cancer, Tumor growth, Tumor angiogenesis, Apoptosis, Hypoxia, PKM2, p65/RelA, HIF-1α, CAM

## Abstract

**Background:**

Initially identified as a molecule that regulates the final step of glycolysis, the M2 isoform of pyruvate kinase (PKM2) was recently reported to have a central role in the metabolic reprogramming of cancer cells as well as participating in cell cycle progression and gene transcription. Despite intensive efforts, the intricate molecular mechanisms through which PKM2 regulates tumor progression remain elusive.

**Methods:**

The proliferation and apoptosis of various pancreatic cancer cells using lentiviral-mediated PKM2 abrogation were assessed *in vitro* via Western blot and flow cytometric assay while the *in vivo* experiments involved tumor xenograft on chicken chorionallantoic membranes and immunohistochemistry on human tissue specimens. In order to decipher the molecular mechanism of HIF-1α and p65/RelA regulation by PKM2 in cancer cells cultivated in hypoxic atmosphere or normoxia we involved various biochemical assays such as Western blotting, immunoprecipitation, reporter gene assay and ELISA.

**Results:**

Strong expression of PKM2 was observed in 68 % of human pancreatic adenocarcinoma specimens and almost all analyzed pancreatic cancer cell lines. Abrogation of PKM2 resulted in impaired proliferation and augmented apoptosis *in vitro* as well as impaired tumor growth and decreased blood vessel formation *in vivo*. Furthermore, deletion of PKM2 negatively impacted hypoxia-induced HIF-1α accumulation and promoter activity ultimately resulting in impaired secretion of VEGF.

**Conclusions:**

Our study suggests that in hypoxic pancreatic tumors PKM2 interferes both with NF-κB/p65 and HIF-1α activation that ultimately triggers VEGF-A secretion and subsequent blood vessel formation.

**Electronic supplementary material:**

The online version of this article (doi:10.1186/s12943-015-0490-2) contains supplementary material, which is available to authorized users.

## Background

The primary function of pyruvate kinase (PK) enzyme is to regulate the final rate-limiting step of glycolysis, which catalyzes the transfer of a phosphate group from phosphoenolpyruvate (PEP) to adenosine disphosphate (ADP), yielding one molecule of pyruvate and one molecule of adenosine triphosphate (ATP) [1]. There are four pyruvate kinase isoenzymes: PKR (expressed in red blood cells), PKL (the liver isoform), PKM1 (found in heart, brain and muscle) and PKM2 (upregulated in embryonic and tumor cells) [2]. PKM1 and PKM2 isoforms are produced upon mutually exclusive alternative splicing of the PKM pre-mRNA, reflecting the inclusion of either exon 9 (*PKM1*) or exon 10 (*PKM2*), respectively [3]. Due to its upregulation in various tumors, PKM2 has garnered attention and was demonstrated to play an essential role in tumor progression [4, 5]. It is worth noting that PKM2 expression levels vary among different types of cancer [6] suggesting that PKM2’s role in tumorigenesis depends on signaling context. Several studies indicate that PKM2 regulates apoptosis and proliferation [7–9]. While siRNA-mediated PKM2 ablation resulted in caspase-mediated apoptosis and tumor regression *in vivo* [10, 11], somatostatin-induced nuclear translocation of PKM2 was associated with the induction of cell death in a caspase-independent manner [8]. A recent view on how elevated levels of PKM2 would benefit proliferating tumor cells is based on the recent findings that PKM2, but not PKM1, can translocate to the nucleus and act both as a protein kinase and as transcriptional coactivator for hypoxia-inducible factor alpha (HIF-1α) in HeLa cervical carcinoma cells [12]. In this study, Luo and colleagues demonstrated that HIF-1α binds hypoxia response elements (HRE) within the first intron of human *PKM2* that contains a HIF-1-binding site (5′-ACGTG-3′) followed by a 5′-CACA-3′ sequence. PKM2 physically interacts with HIF-1α in the nuclei of hypoxic human cancer cells and promotes transactivation of HIF-1α target genes by enhancing the recruitment of p300 to HRE sites [12]. Similarly, phosphoinositide 3-kinase (PI3K) activation has been shown to increase PKM2 expression through HIF-1α-regulated transcription of the *PKM* gene [12, 13]. PKM2 has also been demonstrated to participate in transcriptional activation in response to epidermal growth factor (EGF) [4] and to interact, cooperate with, and be regulated by Oct-4 [9, 14]. Only very recently, PKM2 was reported to interact with NF-κB subunit p65/RelA and to promote tumor angiogenesis and cancer progression [15]. In this study, the authors demonstrated that activation of IGF-1/IGF-1R induces HIF-1α/p65 complex formation, which thus binds to the *PKM* promoter region leading to PKM2 upregulation and PKM2-mediated breast cancer cell growth. Several studies indicated that control of HIF-1α gene by NF-κB provides an important, additional and parallel level of regulation over the HIF-1α pathway [16–19]. Moreover, in the absence of NF-κB, the HIF-1α gene is not transcribed and therefore no stabilization and activity is observed even after prolonged hypoxia [18, 19].

In this study, we investigated the role of PKM2 in angiogenesis of hypoxic pancreatic tumors. We found that PKM2 is expressed in human pancreatic adenocarcinoma and controls VEGF-A secretion by regulating both HIF-1α and NF-κB. Our study favors a signaling mechanism which places the HIF system as a downstream effector of NF-κB biological functions and indicate PKM2 as a kinase that acts upstream of these two transcription factors in hypoxic pancreatic tumors.

## Methods

### Cell lines and reagents

Human pancreatic cancer cell lines used in the study are: Capan1, adenocarcinoma cells derived from pancreatic metastatic site, #ATCC HTB-79; Panc1, a pancreatic epitheloid carcinoma cell line, #ATCC CRL-1469; BxPC3, pancreas adenocarcinoma cells, #ATCC CRL-1687 and Mia Paca-2 carcinoma cells, #ATCC CRL-1420. PaTu2 and PancTu1 pancreatic adenocarcinoma cells were kindly provided by Prof. Simone Fulda, Institute for Experimental Cancer Research in Pediatrics, Frankfurt, Germany. BxPC3 and Capan1 were used for *in vivo* investigations due to their ability to form tumors. Due to higher transient transfection efficacy, PaTu2 and Capan1 were involved in reporter assays and ELISA. Cell lines of early passages were cultured in DMEM (Invitrogen, Germany) supplemented with 10 % fetal calf serum (FCS: Biochrom / Millipore, Germany), 1 % penicillin/streptomycin. BAY 87-2243 was purchased from Seleckchem (#S7309), TEPP-46 was from Millipore (#5.05 487.0001).

### Short hairpins, plasmids, lentiviral transduction and transfection

PKM2-specific shRNAs originate from the “MISSION shRNA Library” designed and developed by the TRC at the Broad Institute of MIT and Harvard. Two PKM2 hairpins (NM_182471.1-1706s1c1- #2 and NM_182471.1-1493s1c1- #4) that showed high efficacy knock-down were selected. The TRC lentiviral human p65/RelA shRNAs were purchased from Thermo Scientific, GE Dharmacon (#RHS4533-EG5970). The most efficient two p65-specific shRNAs (TRCN0000014684-F12 and TRCN0000014687-G3) were used for experiments. The pcDNA3-YFP-p65 expression plasmid was a kind gift from Dr. Franz Oswald, University Hospital of Ulm; pcDNA3-HIF-1α was obtained from Addgene (HIF-1α, #18949). High-titer virus-containing supernatants of HEK293FT cells after transient co-transfection of lentiviral vectors with pMD2.G and psPAX2 packaging vectors were used for lentiviral mediated transduction of cancer cells.

### Promoter assays

Cancer cells plated in 12-well dishes were transfected with 330 ng/ml of the respective promoter plasmid as indicated in the figure legends using Lipofectamine 2000 (Invitrogen #15338-100) or Fugene HD (Promega #E231A) according to the manufacturer instructions. Luciferase activity was determined using the Dual Luciferase Assay Kit (Promega, Mannheim, Germany). Firefly luciferase units were normalized with Renilla luciferase after co-transfection with 17 ng/ml pRL-TK plasmid (Promega, Germany).

### CAM assay

BxPC3 and Capan1 pancreatic cancer cells (1 × 10^6^) transduced with shPKM2 or non-coding shRNA were xenografted within a 5 mm silicon ring on the surface of the chorionallantoic membrane (CAM) of chicken eggs 8 days after fertilization. Four days after implantation (day 12 after fertilization), tumors were retrieved, fixed in formalin and further subjected to immunohistochemistry.

### Immunohistochemistry of CAM and mouse tumors

Formalin-fixed tumors were embedded in paraffin using standard procedures. The 5 μm sections were processed and stained with antibodies directed against Ki67 (1:100; Dako, clone MIB-1), desmin (1:80; Dako, clone D33) and von Willebrand Factor VIII (1:100; Biocare Medical, #CP039B).

### Patient samples and ethics statement

All patient samples had been submitted to the Institutes of Pathology of the Bundeswehrkrankenhaus Ulm or the University Hospital Münster for routine diagnostic purposes between 2003 and 2015. Patient and clinic-pathological data including tumor localization and site of metastasis were obtained from the respective pathology report archives. All samples have been anonymized thoroughly for the use in the study. This study has been approved by the local ethics committee of the University of Münster, Germany (No. 2015-102-f-S).

### Immunohistochemistry and image acquisition of human samples

Immunohistochemistry for PKM2 and CD31 was performed on 4 μm FFPE tissue slides that displayed the invasive margin of the tumors on a Ventana BenchMark Autostainer (Ventana, Tucson, USA) using PKM2 antibody (Abcam, #ab55602, 1:1000) and mouse monoclonal antibody against CD31 (clone JC70, Ventana, Tucson, USA, prediluted) following the manufacturer’s protocol. Light microscopy and image acquisition was performed using a Leica DM6000B light microscope (Leica, Wetzlar, Germany) and the *Diskus Mikroskopische Diskussion* image acquisition software (Carl Hilgers, Königswinter, Germany). PKM2 staining intensity was graded as negative/weak, moderate, or strong.

### Western blot and immunoprecipitation

Whole cell extracts were prepared using IP lysis buffer containing 10 mM Tris-HCl, 5 mM EDTA, 50 mM NaCl, 50 mM NaF and 1 % Triton X100 supplemented with Complete Protease inhibitor Cocktail and PhosStop tablets (Roche). Lysates were subjected to SDS-PAGE and proteins transferred to PVDF membranes (Millipore, Massachusetts, USA). For coIP, protein extracts (0.5 -1.5 mg) were incubated with 2 μg antibody and Protein G-Sepharose (GE Healthcare). Membranes were blocked with 5 % non-fat dry milk in phosphate buffered saline (PBS) containing 0.2 % Tween 20 and incubated over night at 4 °C with specific antibodies. For subsequent washes 0.2 % Tween 20 in PBS was used. The following antibodies were used: PKM2 (Abcam, #ab55602); cleaved PARP (Cell Signaling, #9542S); HIF-1α (BD Transduction Laboratories, #610959); p65/RelA C-20 (Santa Cruz, #sc-372), GFP (Roche, # 1052 1400) and β-actin (Sigma, #A1978). Bands were quantified by densitometric analysis using ImageJ software http://imagej.net.

### FACS analysis

Annexin-V/propidium iodide (PI) staining was performed via flow cytometry according to the manufacturer’s guidelines as previously described [20]. Flow cytometric analysis was performed using FACSCalibur (Becton Dickinson) and the data was analyzed by FlowJo software.

### Hypoxia experiments

The hypoxic environment was generated using the Anaerocult A Mini Kit (Merck, Darmstadt, Germany #1.01611.0001). Cells were incubated under low oxygen atmosphere for 8–24 h at 37 °C.

### Statistics

Statistical analysis was performed with GraphPad Prism 5.0 software (GraphPad Software, Inc, La Jolla, CA, USA). Statistical significance was assessed by an unpaired student *t* test. *p < 0.05* or less was considered significant. The quantitative analysis of immunohistology staining was performed with Image J software (National Institute of Health, Bethesda, MD, USA) using the Colour Deconvolution plugin.

## Results

### PKM2 is highly expressed in human pancreatic tumor specimens

**Fig. 1:**
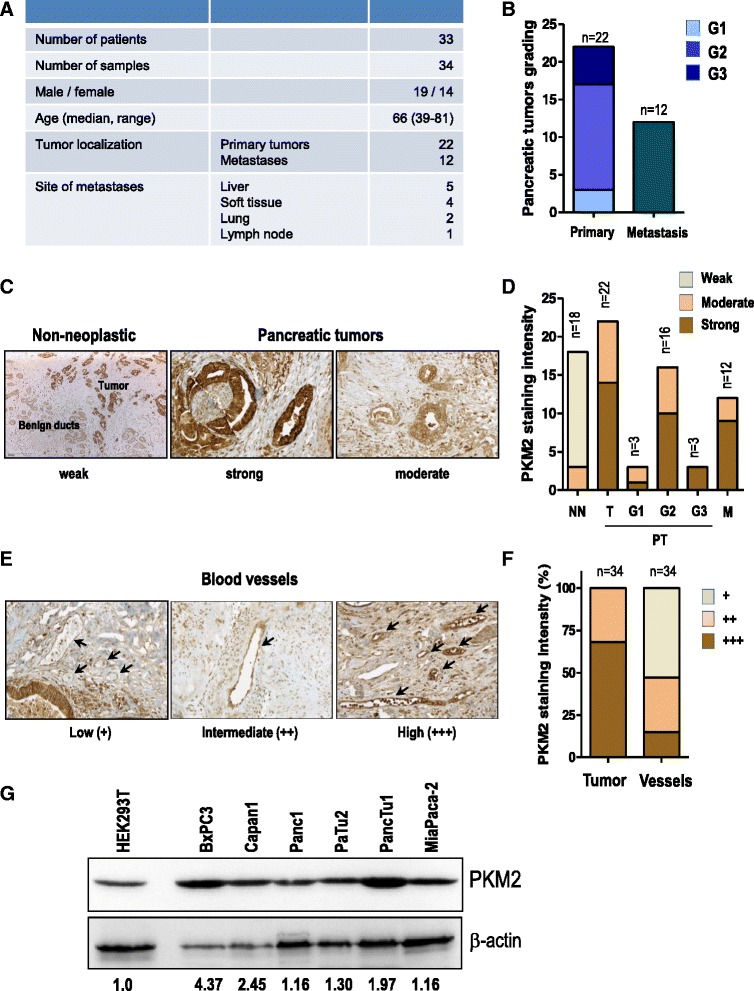
PKM2 is highly expressed in human pancreatic tumor specimens. **a** patient and clinic-pathological data including tumor localization and site of metastasis of the human pancreatic specimens taken into the study are presented. **b** Tumor grading of 34 human pancreatic specimens are shown. **c** PKM2 immunoreactivity was analyzed using standardized protocols. All cancer samples exhibited “moderate” to “strong” PKM2 staining intensity and therefore grouped accordingly. Representative image of each set is depicted. **d** Number of human pancreatic tumors belonging to each grading category and PKM2 immunoreactivity group is presented. **e** PKM2 staining intensity of blood vessels in the same tumor specimens were classified according to immunoreactivity intensity in “high”, “intermediate” and “low”. **f** Bar graph presents the comparison of PKM2 expression levels in tumor-derived blood vessels and human pancreatic tumors. **g** lysates of various pancreatic cancer cell lines were subjected to western blot analysis. Membranes were incubated with PKM2-specific antibody. Quantification of PKM2 band intensity normalized to β-actin was conducted with the ImageJ software. HEK 293 T cells were used as reference (T – tumor; PT – primary tumor; M – metastasis; NN – non-neoplastic tissue; G1 – grade 1; G2 – grade 2 and G3 – grade 3)

PKM2 is expressed in tumors of various tissue origins. We examined the expression of PKM2 by immunohistochemistry in a set of 34 human primary pancreatic ductal adenocarcinomas or metastases to liver, soft tissue, lymph node and lung (Fig. 1a). 63.63 % of the primary tumors were classified as grade 2, 13.63 % grade 1 and 22.72 % grade 3 respectively (Fig. 1b). Virtually all tumor specimens stained positive for PKM2 with 68 % showing strong and 32 % moderate PKM2 immunoreactivity (Fig. 1c-d). PKM2 was not only expressed in the tumor tissue but also in the accompanying vasculature where 15 % of specimens displayed high, 32 % intermediate and 53 % weak expression levels (Fig. 1e-f). This is also supported by our CD31 stainings on human pancreatic cancer specimens showing a dense peritumoral vascular network (Additional file 1: Figure S1). Similar to the pancreatic cancer specimens, all pancreatic adenocarcinoma cancer cell lines investigated expressed elevated levels of PKM2 (Fig. 1g). Altogether, these data suggest that PKM2 may play an important role in pancreatic cancer.

### Effect of PKM2 depletion on tumor cell proliferation and tumor cell viability

**Fig. 2:**
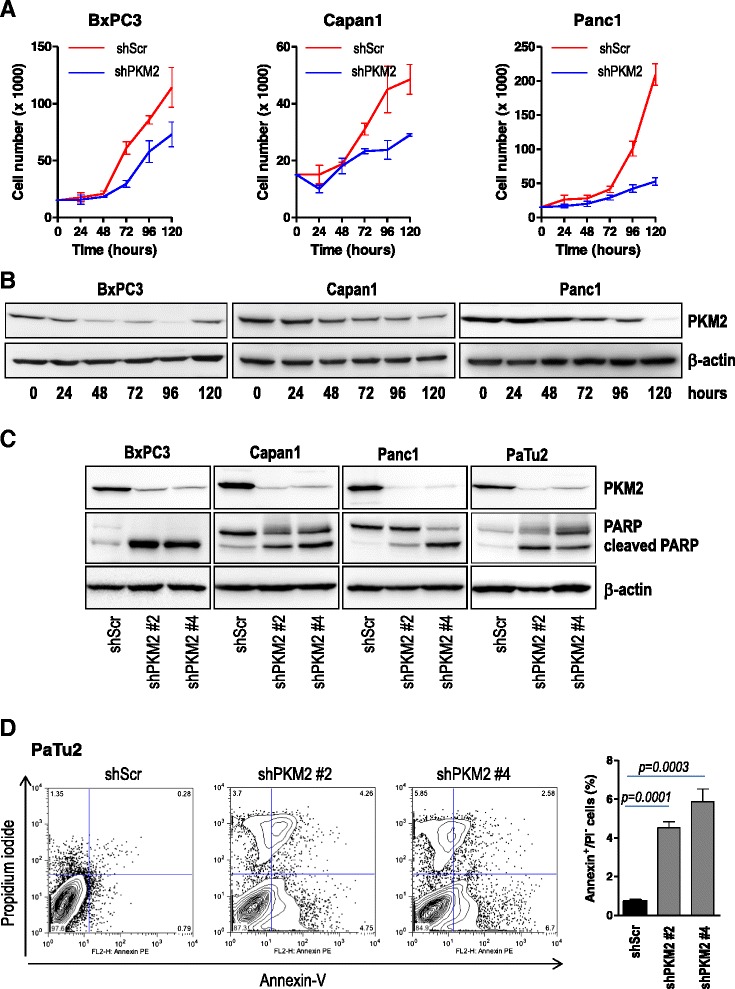
PKM2 depletion is associated with impaired proliferation and augmented apoptosis in tumor cells. **a** 15 × 10^3^ Capan1, BxPC3 and Panc1 pancreatic cancer cells transiently transduced either with a PKM2-specific shRNA or a non-coding shRNA (shScr) were plated in 24 well dishes and allowed to grow for the next 120 h. Cell number was quantified every 24 h. Error bars represent mean +/- SEM of two independent experiments performed in triplicate. **b** lysates of transiently transduced cancer cells were subjected to western blot analysis with PKM2 antibody as indicated. β-actin was used as loading control. **c** various pancreatic cancer cells were stably transduced with two PKM2-specific shRNAs (shPKM2 #2 or shPKM2 #4) or a non-targeting shRNA (shScr). Lysates were prepared and subjected to SDS-PAGE analysis. Membranes were incubated with PKM2 and cleaved PARP antibodies. **d** PaTu2 pancreatic cancer cells stably infected with PKM2-specific shRNAs or a non-targeting shRNA were subjected to annexin V / propidium iodide staining and subsequent flow cytometry. The experiment was performed in triplicate and the data is shown as mean +/- SEM. One of the two experiments is shown

The presence of PKM2 in embryonic tissues is a first sign of its importance for proliferating cells. Cancer cells are highly proliferative and PKM2 has been reported to be important for growth of tumors originating from various tissues such as colon carcinoma, hepatocellular carcinoma and ovarian carcinoma [10]. In order to study the role of PKM2, various pancreatic cancer cell lines were transduced with PKM2-specific shRNAs or a non-targeting shRNA. Abrogation of PKM2 closely correlated with a marked reduction of cell proliferation whereas scrambled (non-targeting) shRNA showed no effect (Fig. 2a and b). To investigate whether PKM2 is crucial for viability of pancreatic cancer cells, we analyzed PARP cleavage by Western blotting. Abrogation of PKM2 in several pancreatic cancer cell lines resulted in augmented cleaved PARP levels (Fig. 2c). We substantiated these results by determination of apoptosis by annexin V/propidium iodide (PI) staining in PaTu2 cancer cells (Fig. 2d).

### PKM2 depletion in pancreatic cancer cells results in impaired tumor growth and angiogenesis *in vivo*

**Fig. 3:**
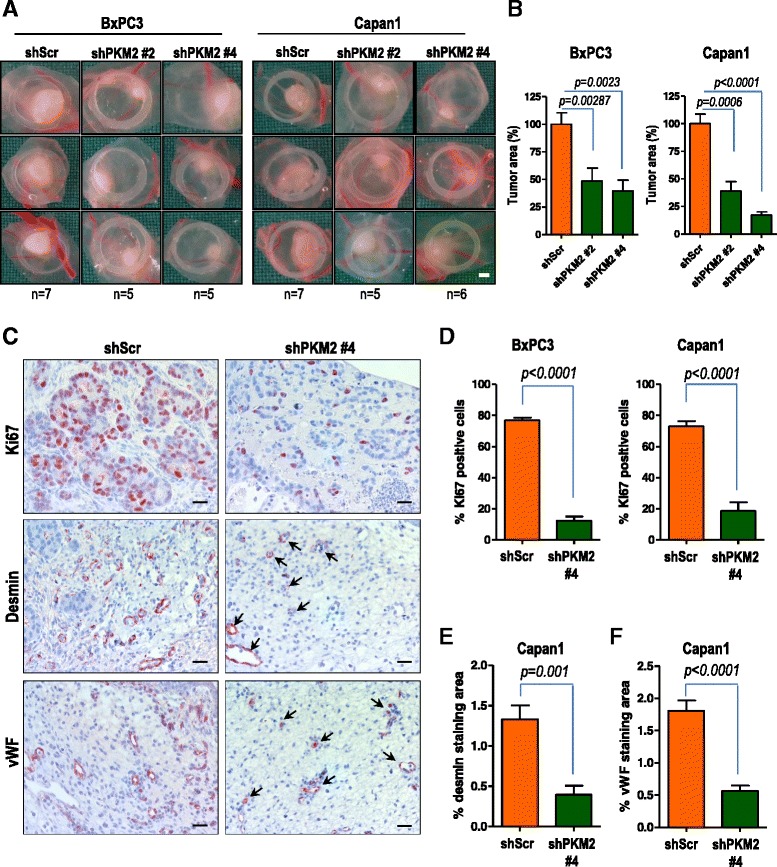
PKM2 abrogation results in impaired tumor growth and decreased blood vessel formation *in vivo*. **a** BxPC3 and Capan1 cells stably transduced with PKM2-specific shRNAs (shKM2 #2 and shPKM2 #4) or a non-coding shRNA (shScr) were delivered to CAM on day 8 after fertilization. Tumor formation was monitored for 96 h. Bar, 1 mm. **b** quantification of tumor area is presented. Error bars represent mean +/- SEM of five to seven tumors. **c** IHC of Capan1 cells growing on CAM using specific antibodies directed against Ki67, desmin and von Willebrand factor (vWF) is presented. Bar, 125 μm. **d** quantification of Ki67 positive BxPC3 or Capan1 cells is shown. Error bars represent mean +/- SEM of at least six microscopic fields, each containing at least 250 cells. **e** desmin and (**f**) vWF immunoreactivities in Capan1 cells were quantified by subtracting background staining from specific signal using Image J software

Having determined that PKM2 is required for pancreatic cancer cell growth *in vitro*, we further evaluated the effect of PKM2 silencing in tumor formation *in vivo* using the chicken chorionallantoic membrane (CAM), an established model of *in vivo* tumor formation [21, 22]. BxPC3 and Capan1 pancreatic cancer cells were transduced with either scrambled oligonucleotide or two PKM2-specific shRNAs and seeded on the surface of CAM eight days after egg fertilization. Four days after implantation, tumors were excised, photographed and analyzed by IHC. BxPC3 and Capan1 control cells formed substantial tumors on the CAM whereas transduction with shRNA targeting PKM2 resulted in significant decrease of tumor size *in vivo* (Fig. 3a and b). This corresponded to significantly reduced proliferation index, as evidenced by the number of Ki67 positive tumor cells (Fig. 3c–d and data not shown). Pancreatic tumors xenografted on chicken CAM trigger formation of an intense *de novo* vascularization, both in the tumor inner core and in the surrounding CAM tissue (Additional file 2: Figure S2). Interestingly, the impaired proliferation of tumor cells transduced with shPKM2 was associated with decreased blood vessel formation of both intratumoral and extratumoral origin/vessels as revealed by reduced immunoreactivity of von Willebrand Factor (vWF) and desmin (Fig. 3c, e–f and Additional file 3: Figure S3).

### Hypoxia induces the nuclear translocation of PKM2 and enhances its interaction with HIF-1α

**Fig. 4:**
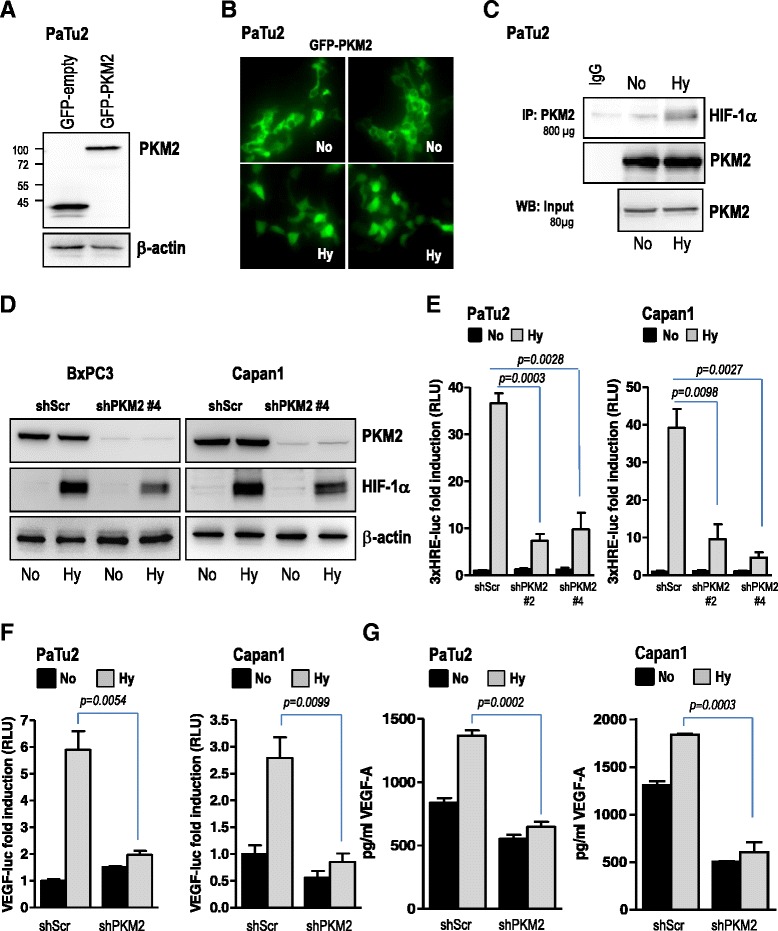
PKM2 regulates hypoxia-induced HIF-1α accumulation and VEGF-A secretion. **a**, **b** PaTu2 cancer cells were transfected with either a GFP-PKM2 construct or GFP empty vector and incubated in normoxic (No) or hypoxic atmosphere (Hy). Forty hours after transfection cells were subjected to immunofluorescence microscopy. **c** immunoprecipitation of endogenous PKM2 was performed with lysates of PaTu2 cultivated in normoxic or hypoxic condition. Membranes were incubated with HIF-1α and PKM2 antibodies. **d** pancreatic cancer cells transduced with a non-targeting control shRNA or PKM2-specific shRNAs were incubated under hypoxia or normoxia for 8 h and HIF-1α levels were determined using western blot analysis. β-actin was used as loading control. **e** pancreatic cancer cells were transiently transfected with 3xHRE-luc and pTK-Renilla and then incubated under normoxic or hypoxic conditions as indicated. Cell lysates were subjected to luciferase assay. Bars are the means +/- SEM of at least three independent experiments. **f** PaTu2 and Capan1 cancer cell lines with abrogated PKM2 were transiently transfected with VEGF-A-luc and pTK-Renilla. Four hours after transfection cells were incubated under normoxic or hypoxic conditions and then cell lysates were subjected to luciferase assay. Bars are the means +/- SEM of at least two independent experiments performed in duplicate. **g** supernatants of pancreatic cancer cells transduced with PKM2-specific shRNAs or a non-coding shRNA incubated in low oxygen were subjected to VEGF-A-specific ELISA. Bars are the means +/- SEM of at least two independent experiments conducted in duplicate

We next sought to investigate the potential mechanism through which PKM2 might regulate blood vessel formation in our experiments presented in Fig. 3. Recently, PKM2 was reported to regulate glucose metabolism by functioning as a coactivator for HIF-1α in cancer cells [12]. Interestingly, we had previously shown that in CAM pancreatic cancer xenografts, augmented tumor vascularization was associated with high expression of HIF-1α in the nuclei [22]. Therefore we wanted to find out whether PKM2 regulates this oxygen sensor molecule. Since gene transactivation is a function of nuclear PKM2, we sought to investigate whether hypoxic environment not only triggers HIF-1α accumulation but also drives the translocation of PKM2 to the nucleus. For these experiments we have utilized a GFP-tagged PKM2 construct (Fig. 4a). Hypoxic cultivation of pancreatic cancer cells transiently expressing GFP-PKM2 resulted in translocation of PKM2 to the nucleus (Fig. 4b). Furthermore, PKM2 translocation was associated with its interaction with hypoxia-stabilized HIF-1α (Fig. 4c), in line with the data presented by Luo and colleagues [12].

### PKM2 knock-down is associated with impaired hypoxia-induced HIF-1α accumulation and VEGF secretion

The nuclear translocation and interaction with HIF-1α posed the question whether PKM2 was able to regulate the expression of this oxygen sensor protein under hypoxic conditions. In our experimental setup using pancreatic tumor cells, abrogation of PKM2 negatively impacted hypoxia-induced accumulation of HIF-1α (Fig. 4d, Additional file 4: Figure S4A-B). Furthermore, impaired HIF-1α accumulation was associated with significant reduction of transcriptional activation of the HIF-responsive element (HRE), a HIF-1α-docking site present in promoters that contain the RCGTG sequence (Fig. 4e). Since upregulation of VEGF-A, an essential driver of blood vessel formation, occurs mainly via stabilization of HIF-1α, we sought to investigate whether PKM2 contributes to VEGF secretion in hypoxic pancreatic cancer cells. ELISA experiments demonstrate that impaired HIF-1α accumulation following depletion of PKM2 was corroborated with impaired low oxygen-driven VEGF promoter activity (Fig. 4f) and decreased levels of hypoxia-induced secreted VEGF-A (Fig. 4g). Same effects were obtained with either TEPP-46, a selective activator molecule reported to force PKM2 into the tetrameric form triggering thus its nuclear exclusion [23] or BAY 87-2243, an HIF-1α-specific inhibitor. Incubation with TEPP-46 or BAY 87-2243 resulted in impaired transcriptional activity of both HIF-1α and VEGF-A in hypoxic pancreatic cancer cells (Additional file 5: Figure S5A–D).

### PKM2 contributes to VEGF secretion in pancreatic cancer cells via activation of NF-κB transcription factors

**Fig. 5:**
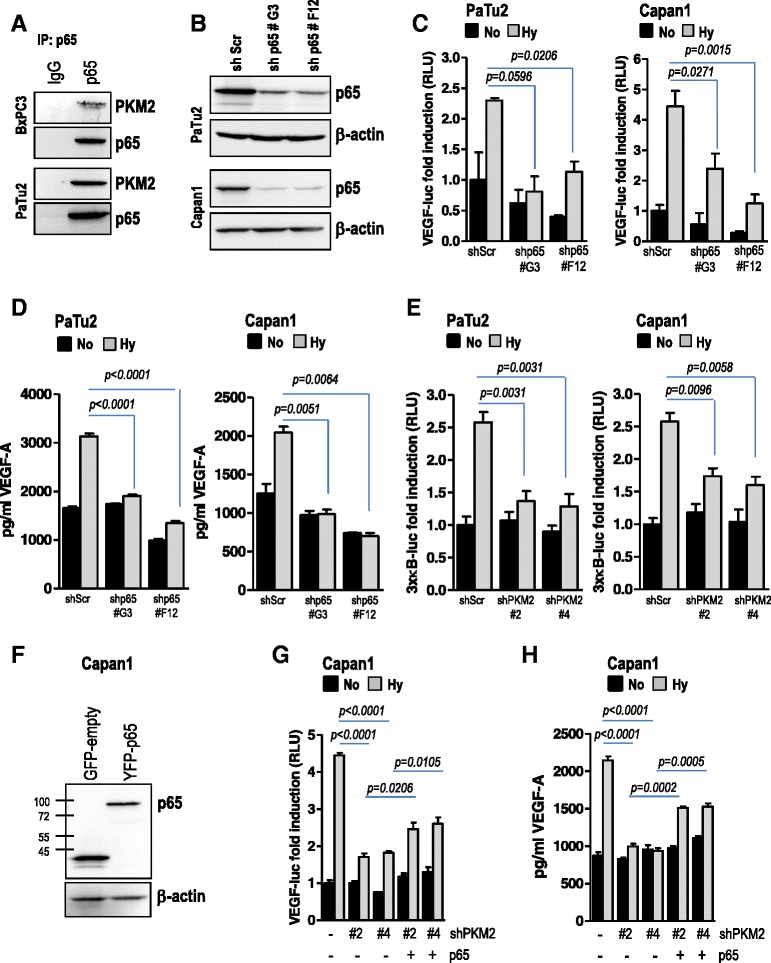
PKM2 mediates hypoxia-triggered VEGF-A secretion by activation of NF-κB/p65 subunit. **a** immunoprecipitation of endogenous p65 was performed with lysates of PaTu2 and BxPC3 cells. Membranes were incubated with PKM2 and reprobed with p65 antibodies. **b**, **c** PaTu2 and Capan1 cancer cell lines with abrogated p65/Rel (shp65 #G3 or shp65 #F12) were transiently transfected with VEGF-A-luc and pTK-Renilla. Four hours later cells were subjected to normoxic or hypoxic conditions. After 24 h cell lysates were prepared and subjected to luciferase assay. Bars are the means +/- SEM of at least three independent experiments. **d** supernatants of PaTu2 or Capan1 cells transduced with p65/RelA-specific shRNA (shp65) or a non-coding shRNA incubated in low oxygen were subjected to VEGF-A-specific ELISA. Bars are the means +/- SEM of at least two independent experiments conducted in duplicate. **e** PaTu2 and Capan1 cancer cell lines with abrogated PKM2 were transiently transfected with 3xκB-luc and pTK-Renilla. After incubation under normoxic or hypoxic conditions cell lysates were prepared and reporter activity assayed. Bars are the means +/- SEM of at least three independent experiments. **f**, **g** pancreatic Capan1 cells with abrogated PKM2 were transiently co-transfected with p65 and VEGF-A-luc reporter before incubation in low oxygen atmosphere. Luciferase was measured after 24 h. **h** supernatants of Capan1 cells with deleted PKM2 and overexpressing p65 cultivated in O_2_-deprived atmosphere or normoxia were subjected to VEGF-A-specific ELISA. Bars are the means +/- SEM of at least two independent experiments conducted in duplicate (No – normoxia; Hy – hypoxia)

The above mentioned data suggest that PKM2 promotes hypoxia-induced VEGF secretion via regulation of HIF-1α. Notably, VEGF-A is not only a target of HIF-1α but also of NF-κB transcription factors. Quite recently, PKM2 was shown to translocate to the nucleus and to interact with p65/NF-κB [15]. Our experiments also show that PKM2 translocates to the nucleus during hypoxia (Fig. 4b) and furthermore, interacts with the NF-κB subunit p65/RelA in pancreatic cancer cells (Fig. 5a), in line with data by Xu and colleagues [15]. We next hypothesized that such an interaction might contribute to VEGF secretion, and therefore depletion of either of PKM2 or p65 would affect VEGF expression. Similar to PKM2, shRNA-mediated depletion of p65/RelA resulted in impaired hypoxia-induced VEGF transcriptional activity (Fig. 5b-c) and decreased hypoxia-stimulated VEGF secretion (Fig. 5d) in pancreatic cancer cells. Interestingly, abrogation of PKM2 was also associated with impaired hypoxia-induced NF-κB transcriptional activity (Fig. 5e) suggesting that PKM2 might regulate VEGF secretion via NF-κB transcription factors. Indeed, ectopic expression of p65/RelA restored hypoxia-induced VEGF promoter activity and secretion after ablation of PKM2 in Capan1 (Fig. 5f-h) and PaTu2 cancer cells (Additional file 6: Figure S6A-C).

### PKM2 regulates HIF-1α -induced VEGF secretion in a NF-κB/p65-dependent manner

**Fig. 6:**
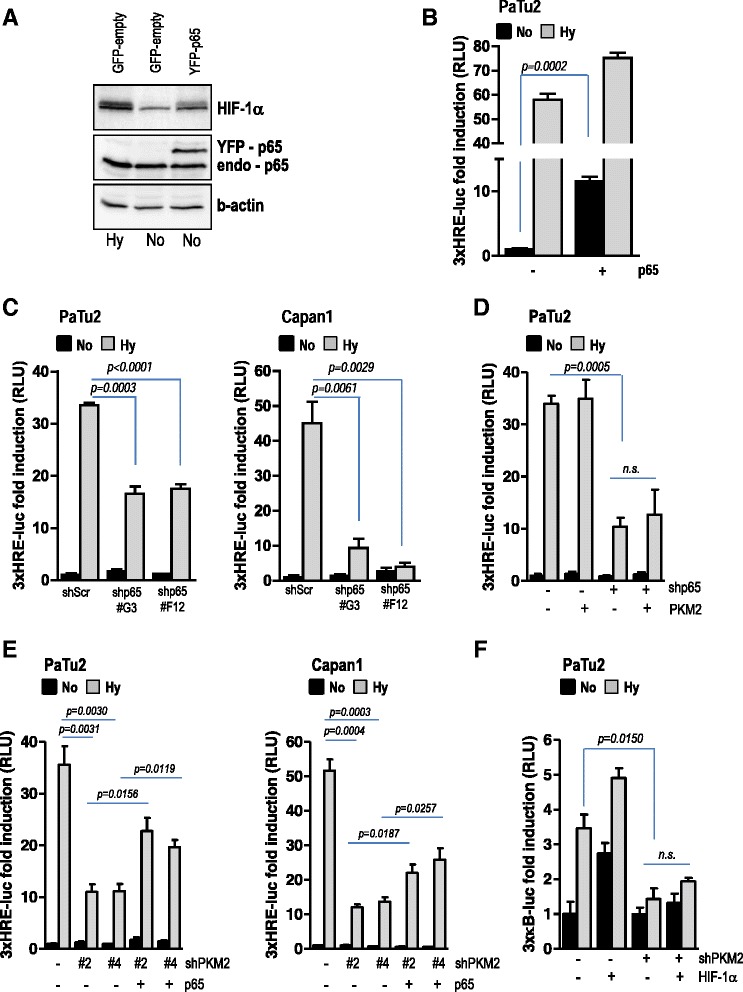
PKM2 regulates HIF-1α transcription via activation of NF-κB signaling pathway. **a** pancreatic cancer cells were transiently transfected with a p65 expression plasmid (YFP-p65). In parallel, cells transfected with control empty vector (GFP-empty) were cultivated in normoxia or hypoxia for 8 h. Lysates were subjected to SDS-PAGE and membranes were incubated with HIF-1α and p65 antibodies. β-actin was used as loading control. **b** PaTu2 cells were co-transfected with p65 and HIF-1α reporter. After incubation for 24 h in low oxygen atmosphere, lysates were prepared and luciferase was measured. **c** cancer cells with abrogated p65 were transfected 3xHRE-luc and then incubated in low oxygen atmosphere. Luciferase was measured 28 h after transfection. Bars are the means +/- SEM of at least three independent experiments. **d** PaTu2 cells featuring p65 knock-down were co-transfected with PKM2 expression plasmid and 3xHRE-luc and then grown in an atmosphere deprived of oxygen. Luciferase was scored 24 h later. Bars are the means +/- SEM of at least three independent experiments. **e** PaTu2 and Capan1 cancer cells with depleted PKM2 were co-transfected with a p65 expression plasmid and 3xHRE-luc and then grown in an atmosphere deprived of oxygen. Luciferase was scored 24 h later. Bars are the means +/- SEM of at least three independent experiments. **f** PaTu2 cells with abrogated PKM2 were co-transfected with a HIF-1α expression plasmid and a 3xκB-luc reporter and then grown in hypoxia. Luciferase was scored 24 h later. Bars are the means +/- SEM of at least three independent experiments. For all reporter assays TK-Renilla was used as internal control

In pancreatic cancer cells, PKM2 appears to regulate hypoxia-induced transcriptional activity of both HIF-1α (Fig. 4e) and NF-κB (Fig. 5e) culminating in VEGF transcription and secretion (Figs. 4f-g; 5g-h and Additional file 6: Figure S6A-C). This suggests that PKM2 participates in the intricate cross-talk between NF-κB and HIF-1α and urged us to elucidate the potential chronology of the signaling events in hypoxic cancer cells leading to VEGF secretion. Belaiba and colleagues demonstrated that in hypoxic cells nuclear NF-κB subunits interact with the κB binding site in the HIF-1α promoter at -197/-188 bp, and thus increase HIF-1α promoter activity and HIF-1α expression [24]. In line with this, we could show that ectopic expression of p65/RelA in pancreatic cancer cells results in augmented HIF-1α accumulation and promoter activity already in normoxic condition (Fig. 6a and b). Conversely, p65 knock-down prevented the activation of HRE in hypoxic cells (Fig. 6c) in line with previous data [18, 19]. Interestingly, overexpression of PKM2 in cancer cells with abrogated p65/RelA had a minimal effect on HIF promoter activity (Fig. 6d). At the same time, ectopic p65/RelA was able to restore hypoxia-induced HIF promoter activity after abrogation of PKM2 in hypoxic pancreatic cancer cells (Fig. 6e). These data suggest that p65 acts upstream of HIF-1α and downstream of PKM2 in pancreatic cancer cells. This hypothesis is supported also by the fact that overexpression of HIF-1α in cells with deleted PKM2 showed only a marginal effect on NF-κB promoter activity (Fig. 6f).

## Discussion

The primary function of pyruvate kinase M2 isoform (PKM2) is to catalyze the phosphorylation from phosphoenolpyruvate (PEP) to pyruvate as the last step of glycolysis to generate ATP. Incidentally, tumor cells need a permanent supply of glycolytic intermediates [25, 26] and an impaired activity of PKM2 (through dimer formation) was observed to favor rapid cell proliferation. Several studies from recent years demonstrated that PKM2 may play a far broader role in promoting cancer progression than has previously been appreciated. However, the intricate molecular mechanisms through which PKM2 exerts its tumorigenic role remain elusive.

In this study, we have identified PKM2 as a crucial molecule for progression of pancreatic cancer in which the tumor microenvironment has been reported to be highly hypoxic [27]. Accordingly, we found moderate to strong PKM2 expression in all examined human pancreatic adenocarcinoma samples. Depletion of PKM2 was associated with impaired proliferation and augmented tumor cell death *in vitro*, while the *in vivo* tumor xenograft experiments revealed a close association between impaired tumor growth and decreased blood vessel formation. These findings, together with earlier studies showing that PKM2 promotes angiogenesis by binding to TEM8 - tumor endothelial factor 8 [28], suggest that PKM2 might contribute to tumor growth by regulating crucial molecules involved in tumor vascularization. Such a molecule is the well characterized VEGF. The fact that VEGF is directly regulated by HIF-1α, an oxygen sensor protein reported to interact with PKM2 [12] urged us to evaluate the role of PKM2 with respect to HIF-1α stabilization and VEGF secretion in hypoxic pancreatic tumors. Firstly, we showed that PKM2 translocates to the nucleus in pancreatic cancer cells cultivated in hypoxic conditions. Secondly, the interaction between PKM2 and HIF-1α is augmented in hypoxic cancer cells. Lastly, abrogation of PKM2 prevented hypoxia-mediated HIF-1α accumulation and HIF-1α promoter activity, which negatively impacted VEGF secretion by pancreatic cancer cells deprived of oxygen. Together, these findings suggest that PKM2 is required for HIF-1α mediated VEGF transcriptional activity and secretion. Hypoxia not only results in the accumulation of HIF-1α, but also activates the NF-κB transcription factors [29]. Because PKM2 was reported to directly interact with the NF-κB p65 subunit to promote EGR1 expression [15], we explored the possibility whether PKM2 interferes with NF-κB activation by hypoxia. Indeed, PKM2 expression arrest was mirrored by impaired hypoxia-driven promoter activity of NF-κB and its target gene VEGF. Ectopic expression of p65 restored VEGF transcription after PKM2 ablation inferring that the kinase regulates VEGF via NF-κB p65 subunit. This finding is also supported by the fact that PKM2 interacts with p65 in pancreatic cancer cells. It has been previously shown that activation of NF-κB and transcript abundance of p65 are diminished in HIF-1α-deficient mice [30] and that the NF-κB subunits translocated to the nucleus interact with the NF-κB binding sites in the HIF-1α promoter, thus increasing HIF-1α promoter activity and expression [18, 24]. The fact that HIF-1α itself contributes to the activation of the NF-κB pathway adds a further level of complexity to the cross-talk between these molecules. Our data demonstrate that PKM2 is able to regulate the NF-κB as well as the HIF-1α signaling pathways and suggest that the kinase acts upstream of these two transcription factors in pancreatic tumors. Interestingly, ectopic expression of p65 restored HIF-1α transcriptional activity in hypoxic cells featuring PKM2 knock-down. In the same cells however, overexpression of HIF-1α showed only marginal effect on NF-κB transcription. While ectopic expression of p65/RelA resulted in augmented HIF-1α transcription already in normoxic condition, the overexpression of PKM2 had no impact on HIF-1 promoter activity in cells with abrogated p65. These data place the NF-κB p65 subunit below PKM2 and upstream of HIF-1α in the hypoxia signaling axis.

## Conclusion

**Fig. 7:**
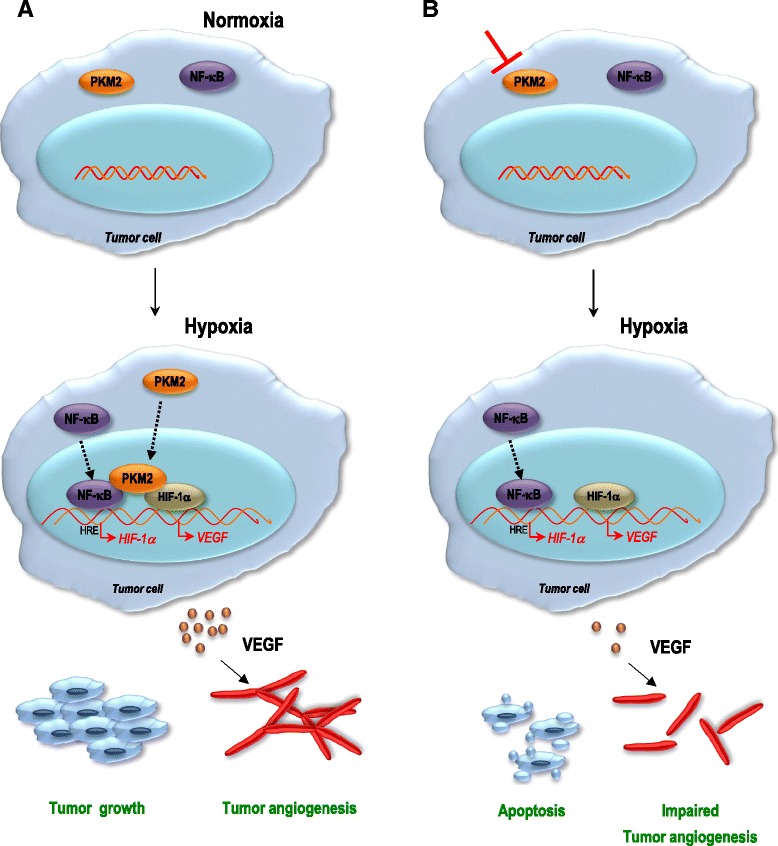
PKM2 contributes to tumor growth and angiogenesis by regulating HIF-1α via NF-κB activation. **a** in physiological conditions PKM2 and p65 reside in the cytosol. Hypoxic insult is followed by translocation of PKM2 and p65 to the nucleus in pancreatic cancer cells. Following the interaction with PKM2, NF-κB subunit p65 activates the transcription of HIF-1α and its target gene VEGF-A. As a result, augmented secretion of VEGF translates to a boost in blood vessel formation, which in turn contributes to tumor growth. **b** in the absence of PKM2, transcription and secretion of VEGF-A is still present, yet to a lower level. While our data do not exclude the VEGF secretion as a result of sole NF-κB activation *or* HIF-1α accumulation, they emphasize the essential role of PKM2 in the regulation of HIF-1α and NF-κB transcription factor during tumor angiogenesis and tumor growth of hypoxic pancreatic tumors

In conclusion, our data favor a scenario where the hypoxic insult/intratumoral hypoxia is followed by translocation of PKM2 and p65 to the nucleus in pancreatic cancer cells. Following the interaction, NF-κB subunit p65 activates the transcription of *HIF-1α* and its target genes, such as *VEGF-A*. As a result, augmented secretion of VEGF translates to a boost in blood vessel formation that in turn contributes to tumor growth (Fig. 7). Since VEGF is a target of both HIF-1α *and* NF-κB, we cannot exclude the contribution of VEGF secretion as a result of sole NF-κB activation *or* HIF-1α accumulation in other tumor entities. We also think that basal HIF-1α protein levels are critical for the cell’s readiness to respond to hypoxia, and therefore render NF-κB to be critical for the degree and speed towards HIF-1α activation after a hypoxic onslaught.

Since in human pancreatic ductal adenocarcinoma (PDAC) HIF-1α expression highly correlates with tumor size and poor prognosis [31] it will be a demanding task to dissect the different signaling states and the potential of feed-back signaling loops which may be of great importance for therapeutic targeting of pancreatic tumors using PKM2 inhibitors currently under intense development.

## Additional files

Additional file 1: Figure S1. 34 human pancreatic specimens were stained with PKM2 and CD31 antibodies. Representative images are shown. (PPTX 760 kb) Additional file 2: Figure S2. IHC of pancreatic cancer cells growing on CAM using specific antibodies directed against desmin and von Willebrand factor (vWF) is presented. The images reveal both intratumoral and extratumoral immunoreactivity of the endothelial markers. (PPTX 4480 kb) Additional file 3: Figure S3. PKM2 abrogation results in decreased blood vessel formation *in vivo*. IHC of BxPC3 pancreatic cancer cells growing on CAM using specific antibodies for desmin and von Willebrand factor (vWF) is presented. (PPTX 3897 kb) Additional file 4: Figure S4. PKM2 regulates hypoxia-induced HIF-1α accumulation. A, B, pancreatic cancer cells transduced with a non-targeting control shRNA or PKM2-specific shRNAs were incubated under hypoxia or normoxia for 8 h. HIF-1α levels were determined using western blot analysis. Quantification of HIF-1α band intensity was conducted using ImageJ software (No – normoxia; Hy – hypoxia). (PPTX 72 kb) Additional file 5: Figure S5. PKM2 regulates hypoxia-induced VEGF promoter activity. A, PaTu2 cancer cells were transiently transfected with 3xHRE-luc and pTK-Renilla. Four hours after transfection cells were incubated under normoxic or hypoxic conditions in the absence or presence of 30 μM TEPP-46. Cell lysates were subjected to luciferase assay. Bars are the means +/- SEM of at least two independent experiments performed in duplicate. B, cancer cells were transiently transfected with VEGF-luc and pTK-Renilla. Four hours after transfection cells were incubated under normoxic or hypoxic conditions in the absence or presence of 30 μM TEPP-46. Cell lysates were subjected to luciferase assay. Bars are the means +/- SEM of at least two independent experiments performed in duplicate. C, D, PaTu2 and Capan1 cells were transiently transfected with VEGF-luc reporter. Four hours later cells were incubated under normoxic or hypoxic conditions in the absence or presence of 10 μM BAY 87-2243. Lysates were subjected to luciferase assay. Bars are the means +/- SEM of at least two independent experiments performed in duplicate (No – normoxia; Hy – hypoxia). (PPTX 138 kb) Additional file 6: Figure S6. PKM2 mediates hypoxia-triggered VEGF-A secretion by activation of NF-κB/p65 subunit. A, western blot showing the expression of YFP-p65 in PaTu2 pancreatic cancer cells is presented. B, PaTu2 cancer cells with abrogated PKM2 were transiently co-transfected with p65 expression plasmid and VEGF-A-luc reporter before incubation in low oxygen atmosphere. Luciferase was measured after 24 h. C, supernatants of PaTu2 cells with deleted PKM2 and overexpressing p65 cultivated in O_2_-deprived atmosphere or normoxia were subjected to VEGF-A-specific ELISA. Bars are the means +/- SEM of at least two independent experiments conducted in duplicate (No – normoxia; Hy – hypoxia). (PPTX 119 kb)
