# Supplementary figures and images for: PKM2 promotes tumor angiogenesis by regulating HIF-1α through NF-κB activation
Source: Mol Cancer. 2016 Jan 6;15:3. doi: 10.1186/s12943-015-0490-2 (PMC4704385; doi:10.1186/s12943-015-0490-2)

## Slide 1
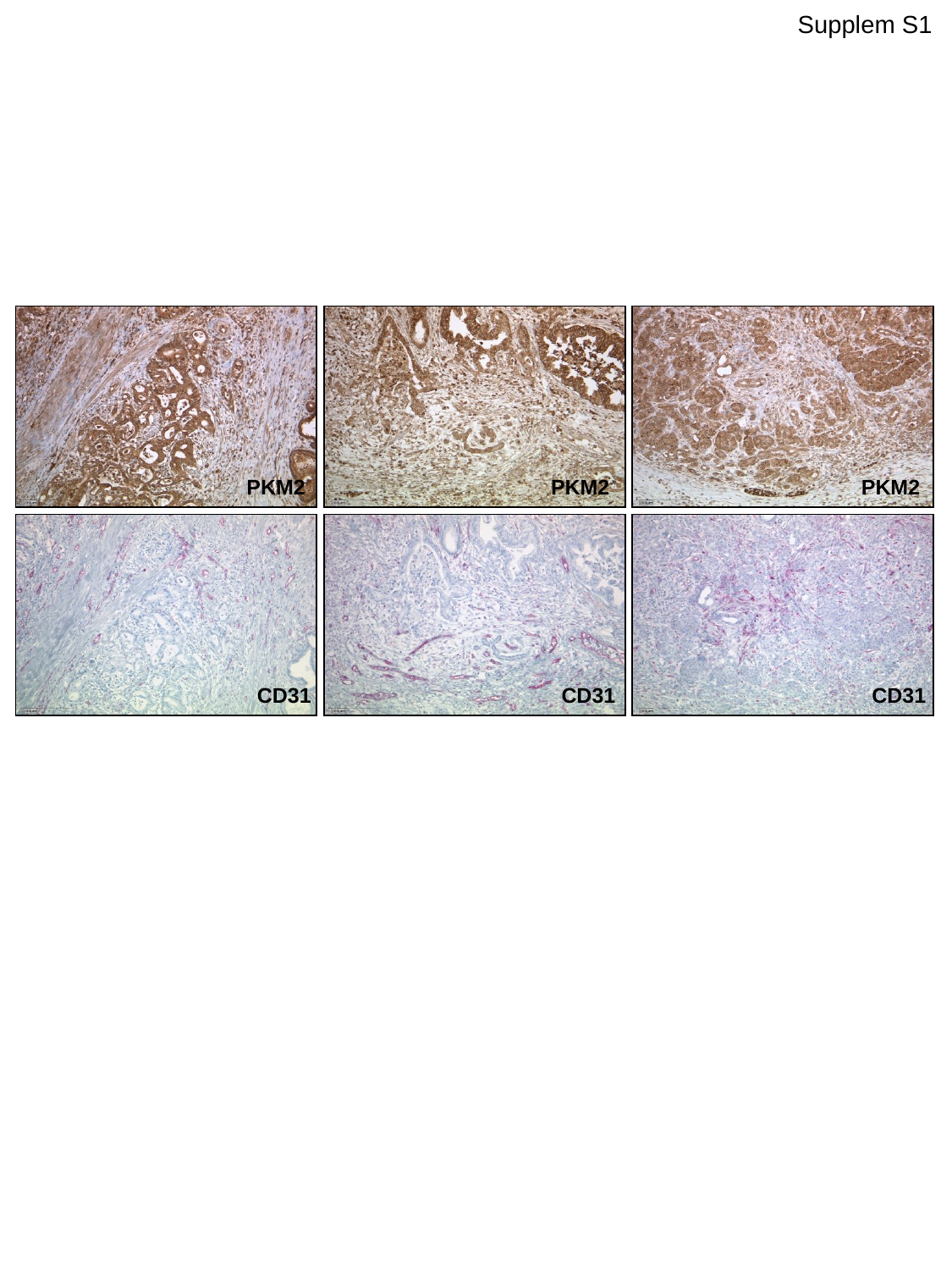

Supplem S1
PKM2
PKM2
PKM2
CD31
CD31
CD31

Supplement: Additional file 1: Figure S1. — 34 human pancreatic specimens were stained with PKM2 and CD31 antibodies. Representative images are shown. (PPTX 760 kb) [file 12943_2015_490_MOESM1_ESM.pptx]

## Slide 1
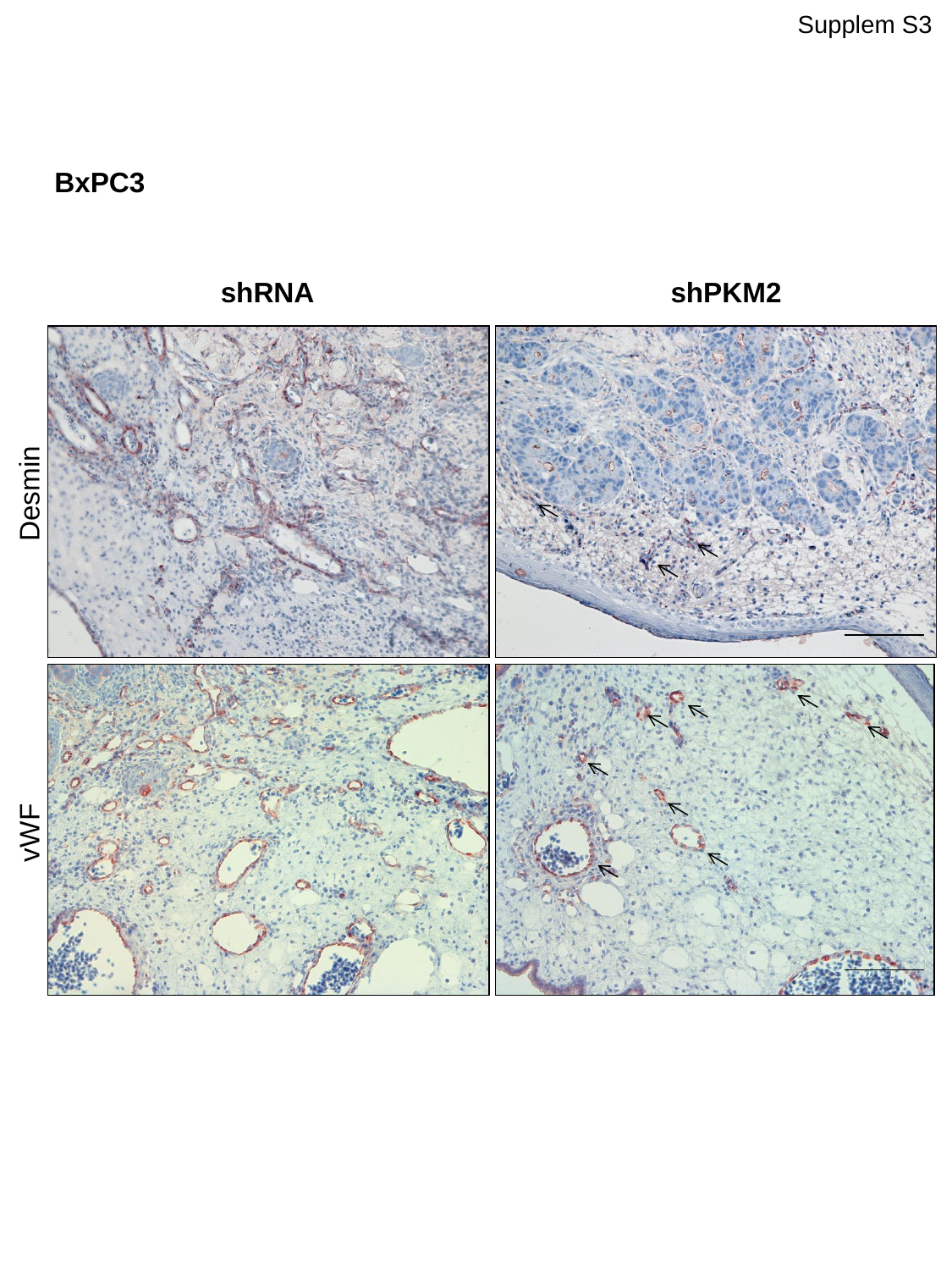

Supplem S3
BxPC3
shRNA
shPKM2
Desmin
vWF

Supplement: Additional file 3: Figure S3. — PKM2 abrogation results in decreased blood vessel formation in vivo. IHC of BxPC3 pancreatic cancer cells growing on CAM using specific antibodies for desmin and von Willebrand factor (vWF) is presented. (PPTX 3897 kb) [file 12943_2015_490_MOESM3_ESM.pptx]

## Slide 1
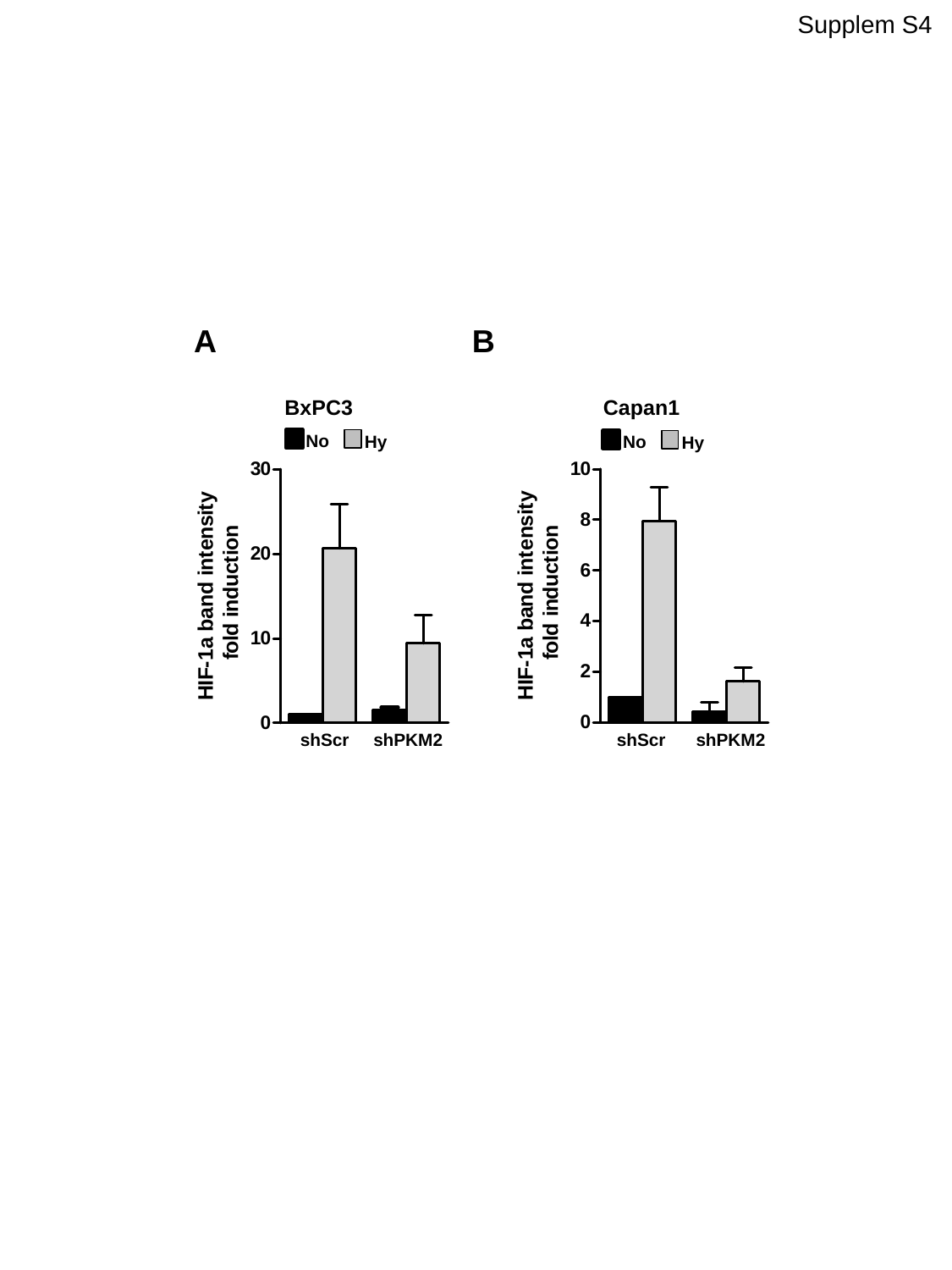

Supplem S4
A
B
BxPC3
Capan1
No
No
Hy
Hy
shScr
shPKM2
shScr
shPKM2

Supplement: Additional file 4: Figure S4. — PKM2 regulates hypoxia-induced HIF-1α accumulation. A, B, pancreatic cancer cells transduced with a non-targeting control shRNA or PKM2-specific shRNAs were incubated under hypoxia or normoxia for 8 h. HIF-1α levels were determined using western blot analysis. Quantification of HIF-1α band intensity was conducted using ImageJ software (No – normoxia; Hy – hypoxia). (PPTX 72 kb) [file 12943_2015_490_MOESM4_ESM.pptx]

## Slide 1
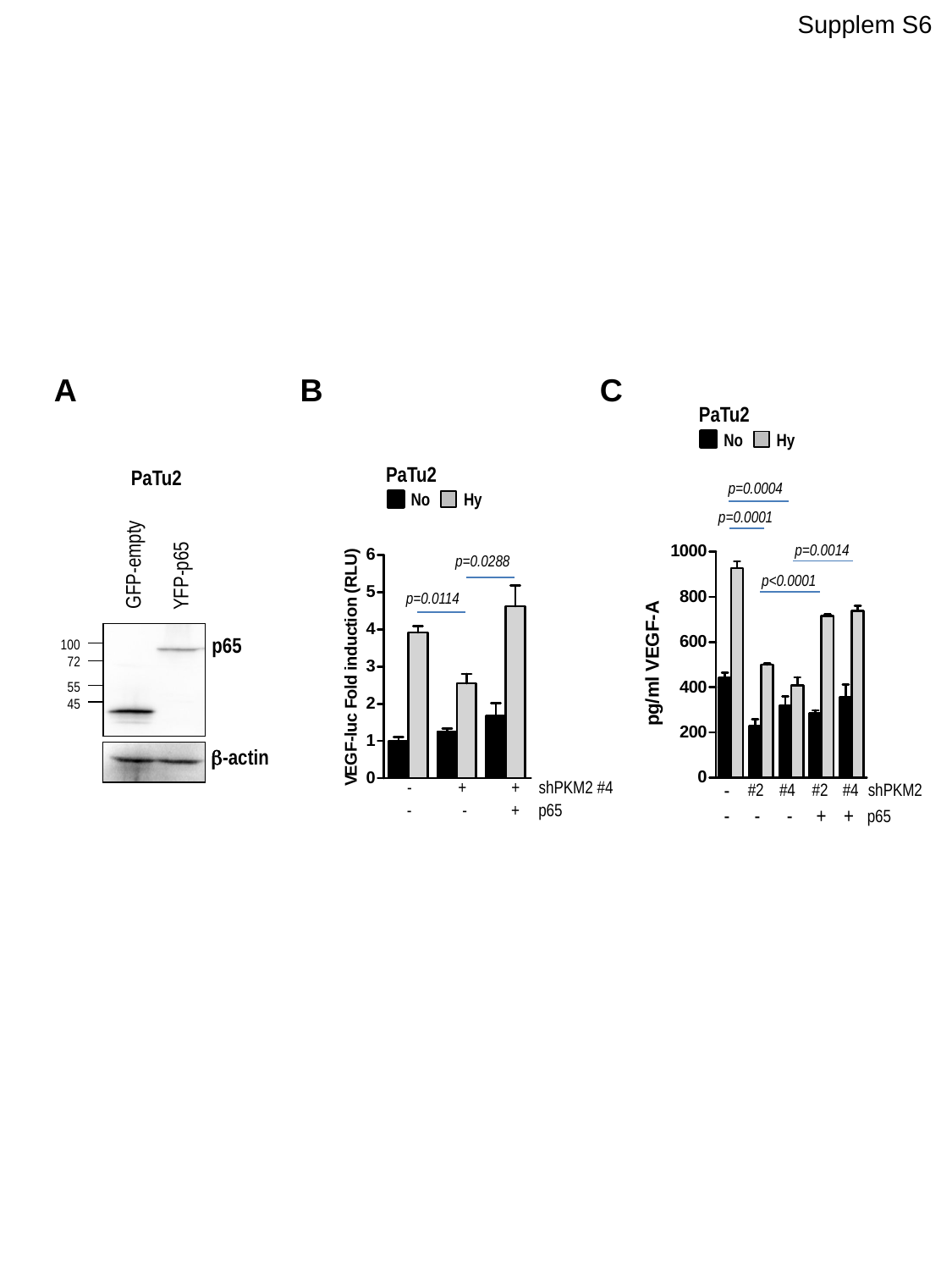

Supplem S6
A
B
C
PaTu2
No
Hy
p=0.0004
p=0.0001
p=0.0014
p<0.0001
-
#2
#4
#2
#4
shPKM2
-
-
-
+
+
p65
PaTu2
No
Hy
p=0.0288
p=0.0114
-
+
+
shPKM2 #4
-
-
+
p65
PaTu2
GFP-empty
YFP-p65
p65
100
72
55
45
b-actin

Supplement: Additional file 6: Figure S6. — PKM2 mediates hypoxia-triggered VEGF-A secretion by activation of NF-κB/p65 subunit. A, western blot showing the expression of YFP-p65 in PaTu2 pancreatic cancer cells is presented. B, PaTu2 cancer cells with abrogated PKM2 were transiently co-transfected with p65 expression plasmid and VEGF-A-luc reporter before incubation in low oxygen atmosphere. Luciferase was measured after 24 h. C, supernatants of PaTu2 cells with deleted PKM2 and overexpressing p65 cultivated in O2-deprived atmosphere or normoxia were subjected to VEGF-A-specific ELISA. Bars are the means +/- SEM of at least two independent experiments conducted in duplicate (No – normoxia; Hy – hypoxia). (PPTX 119 kb) [file 12943_2015_490_MOESM6_ESM.pptx]
