# Supplementary material for: PKM2 promotes tumor angiogenesis by regulating HIF-1α through NF-κB activation
Source: Mol Cancer. 2016 Jan 6;15:3. doi: 10.1186/s12943-015-0490-2 (PMC4704385; doi:10.1186/s12943-015-0490-2)
Supplement: Additional file 2: Figure S2. — IHC of pancreatic cancer cells growing on CAM using specific antibodies directed against desmin and von Willebrand factor (vWF) is presented. The images reveal both intratumoral and extratumoral immunoreactivity of the endothelial markers. (PPTX 4480 kb) [file 12943_2015_490_MOESM2_ESM.pptx]

## Slide 1
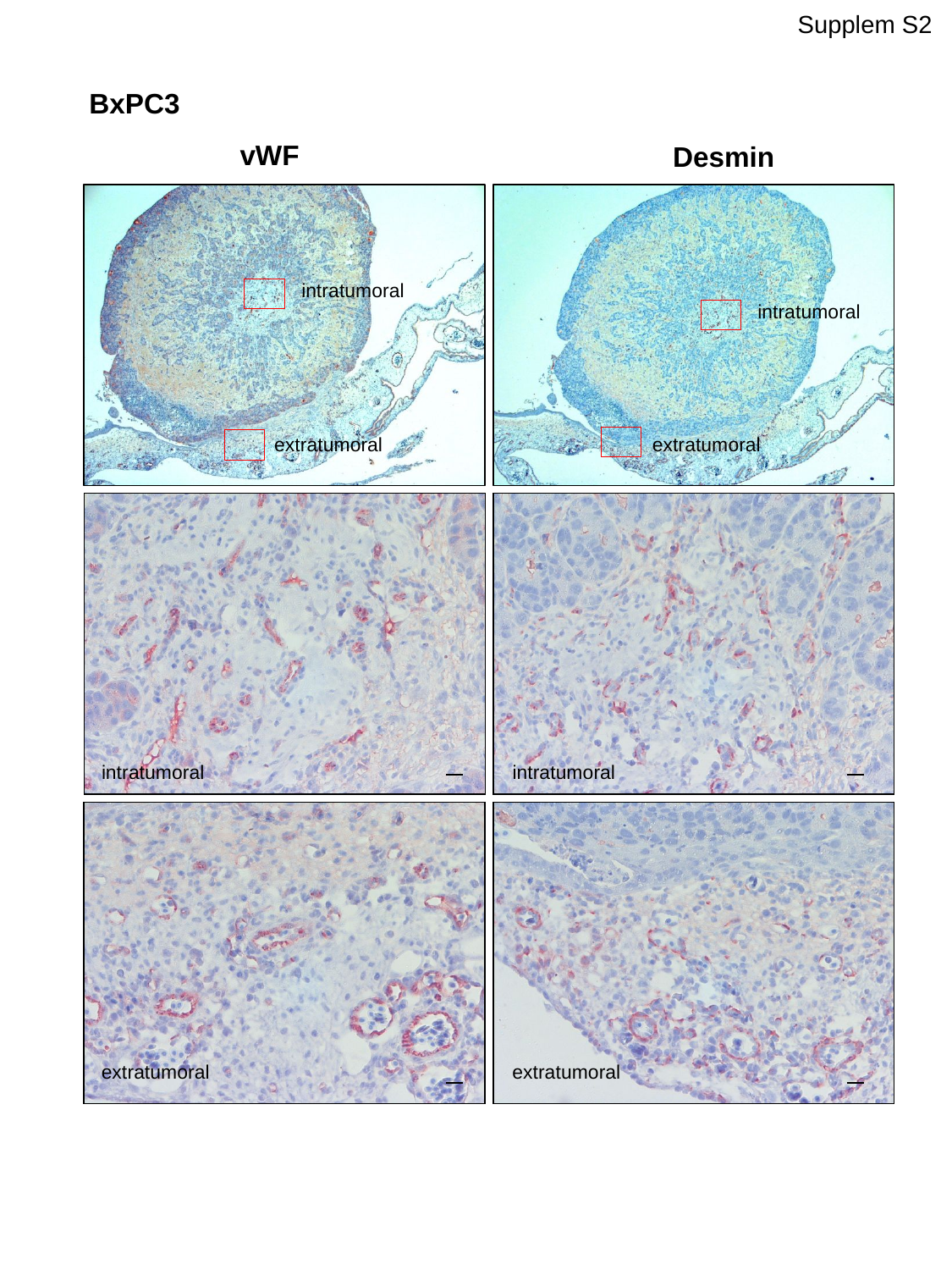

Supplem S2
BxPC3
vWF
Desmin
intratumoral
intratumoral
extratumoral
extratumoral
intratumoral
intratumoral
extratumoral
extratumoral
