# Supplementary material for: PKM2 promotes tumor angiogenesis by regulating HIF-1α through NF-κB activation
Source: Mol Cancer. 2016 Jan 6;15:3. doi: 10.1186/s12943-015-0490-2 (PMC4704385; doi:10.1186/s12943-015-0490-2)
Supplement: Additional file 5: Figure S5. — PKM2 regulates hypoxia-induced VEGF promoter activity. A, PaTu2 cancer cells were transiently transfected with 3xHRE-luc and pTK-Renilla. Four hours after transfection cells were incubated under normoxic or hypoxic conditions in the absence or presence of 30 μM TEPP-46. Cell lysates were subjected to luciferase assay. Bars are the means +/- SEM of at least two independent experiments performed in duplicate. B, cancer cells were transiently transfected with VEGF-luc and pTK-Renilla. Four hours after transfection cells were incubated under normoxic or hypoxic conditions in the absence or presence of 30 μM TEPP-46. Cell lysates were subjected to luciferase assay. Bars are the means +/- SEM of at least two independent experiments performed in duplicate. C, D, PaTu2 and Capan1 cells were transiently transfected with VEGF-luc reporter. Four hours later cells were incubated under normoxic or hypoxic conditions in the absence or presence of 10 μM BAY 87-2243. Lysates were subjected to luciferase assay. Bars are the means +/- SEM of at least two independent experiments performed in duplicate (No – normoxia; Hy – hypoxia). (PPTX 138 kb) [file 12943_2015_490_MOESM5_ESM.pptx]

## Slide 1
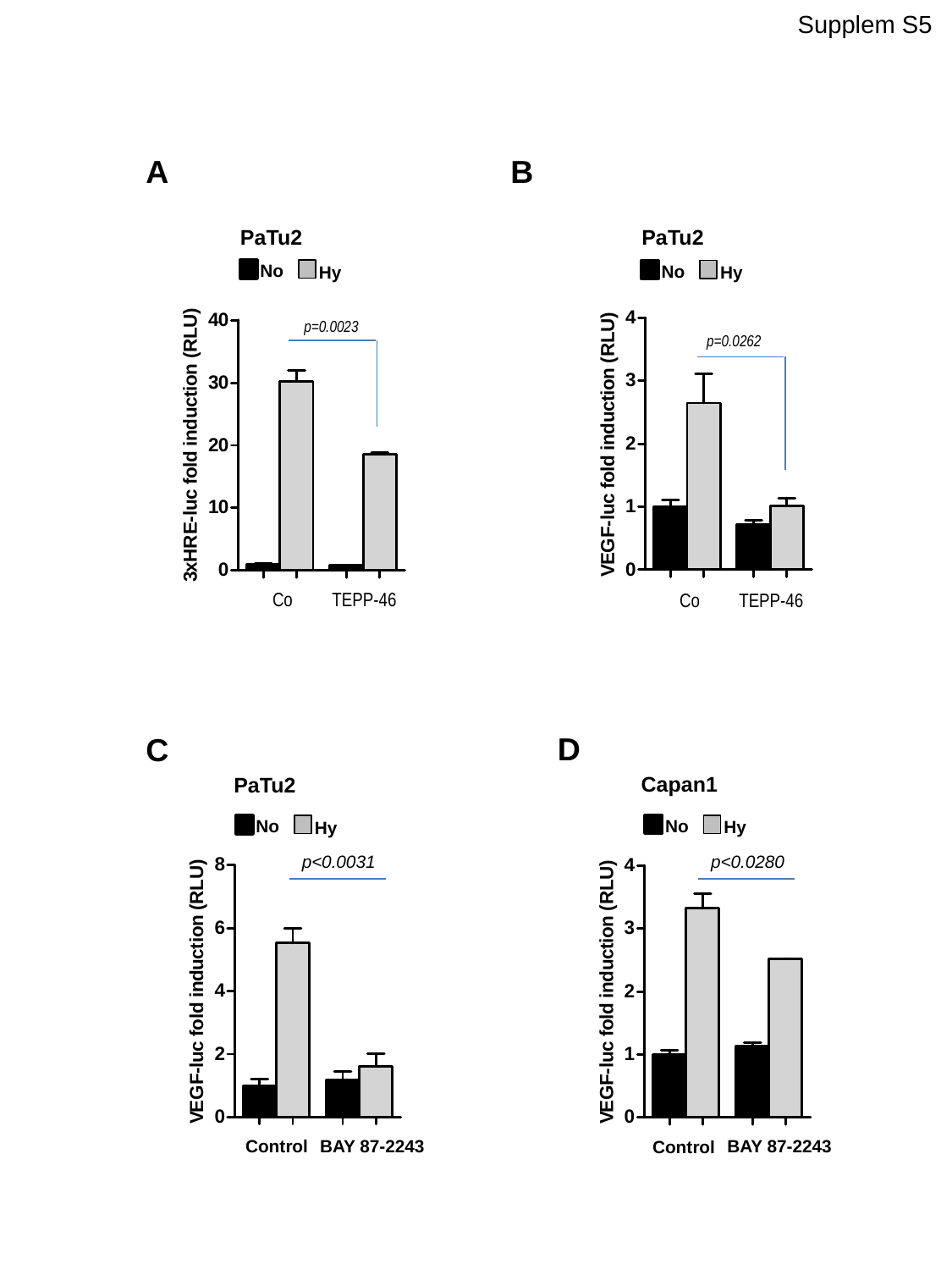

Supplem S5
A
B
PaTu2
PaTu2
No
No
Hy
Hy
p=0.0023
p=0.0262
Co
TEPP-46
Co
TEPP-46
D
C
Capan1
PaTu2
No
No
Hy
Hy
p<0.0031
p<0.0280
BAY 87-2243
Control
BAY 87-2243
Control
